# Supplementary material for: Overcrowding induces fast colloidal solitons in a slowly rotating potential landscape
Source: Nat Commun. 2023 Oct 13;14:6448. doi: 10.1038/s41467-023-41989-x (PMC10575966; doi:10.1038/s41467-023-41989-x)
Supplement: Supplementary file 1 — Supplementary Information File [file 41467_2023_41989_MOESM1_ESM.pdf]

Supplementary Information for
Overcrowding induces fast colloidal solitons in a slowly rotating potential landscape

Eric Cereceda-López,[1,2] Alexander P. Antonov,[3] Artem Ryabov,[4] Philipp Maass,[3] and Pietro Tierno[1,2,5]

[1] *Departament de Física de la Matèria Condensada, Universitat de Barcelona, 08028, Spain*
[2] *Institut de Nanociència i Nanotecnologia, Universitat de Barcelona (IN2UB), 08028, Barcelona, Spain*
[3] *Universität Osnabrück, Fachbereich Physik, Barbarastraße 7, D-49076 Osnabrück, Germany*
[4] *Charles University, Faculty of Mathematics and Physics, Department of Macromolecular Physics, V Holešovičkách 2, CZ-18000 Praha 8, Czech Republic*
[5] *University of Barcelona Institute of Complex Systems (UBICS), 08028, Barcelona, Spain*

SUPPLEMENTARY DISCUSSION 1. DYNAMICS IN THE ZERO-NOISE LIMIT

Suppl. Note 1. Equations of motions and rescaled variables

In the zero-noise limit, the system dynamics is deterministic and periodic. For calculating features of the soliton dynamics, we consider the traveling-wave potential $U(x,t) = (U_0/2)\cos[2\pi/\lambda(x+\omega R\,t)]$ with $x = R\varphi$, $\lambda = 2\pi R/M$ along the ring of the M optical traps. When $|x_i - x_j| > \sigma$, the equations of motion for the particle coordinates $x_i = R\varphi_i$ are

$$\frac{\mathrm{d}x_i}{\mathrm{d}t} = -\mu\frac{\partial}{\partial x_i}\left(\frac{U_0}{2}\cos\left[\frac{2\pi}{\lambda}(x_i+\omega R\,t)\right]\right) = \mu\frac{\pi U_0}{\lambda}\sin\left[\frac{2\pi}{\lambda}(x_i+\omega R\,t)\right]\,. \tag{1}$$

Transforming these equations to the corotating frame, where particle have coordinates $x_i' = x_i + \omega R\,t$, we obtain

$$\frac{\mathrm{d}x_i'}{\mathrm{d}t} = \omega R + \frac{\mu U_0\pi}{\lambda}\sin\left(\frac{2\pi x_i'}{\lambda}\right) = \mu F^{\mathrm{ext}}(x_i')\,. \tag{2}$$

Here, $F^{\mathrm{ext}}(x')$ is the time-independent force in the corotating frame.

When particles come into contact, the force conditions given by Eq. (2) in the main text must be taken into account as well as the fact that n particles with coordinates $x_1', x_2' = x_1' + \sigma, \ldots, x_n' = x_1' + (n-1)\sigma$ moving together have a velocity $v_n'(x_1') = \mu\bar{F}^{\mathrm{ext}}(x_1') = v_n(x_1) + \omega R$, where $\bar{F}_n^{\mathrm{ext}}(x_1') = \sum_{j=1}^{n} F^{\mathrm{ext}}(x_1' + (j-1)\sigma)/n$ is mean force on the cluster. Here and in the following we specify the position of an n-cluster by the position of its leftmost particle.

Introducing scaled coordinates and time, and a rescaling of the angular velocity,

$$x_i' \to y_i = \frac{x_i'}{\lambda}\,, \quad t \to \frac{\lambda^2}{\pi\mu U_0}t\,, \quad \omega R \to f = \frac{\lambda}{\mu\pi U_0}\omega R\,, \tag{3}$$

Eqs. (2) take the form

$$\frac{\mathrm{d}y_i}{\mathrm{d}t} = f + \sin(2\pi y_i)\,, \tag{4}$$

where $f < 1$ for undercritical tilting of the potential, i.e. we consider $0 \le f < 1$. The hard sphere constraints between the y coordinates is given by the rescaled hard sphere diameter

$$\sigma \to \frac{\sigma}{\lambda} < 1\,, \tag{5}$$

and velocities are rescaled according to

$$v' \to u = \frac{\lambda}{\mu\pi U_0}\,v'\,. \tag{6}$$

The positions of mechanical equilibrium for a single particle are

$$y_p^{\mathrm{eq}} = \frac{1}{2} + \frac{1}{2\pi}\arcsin(f) + p\,, \quad p = 0, \ldots, M-1\,, \tag{7}$$

where $\arcsin(.) \in [-\pi/2, \pi/2]$.

Suppl. Note 2. Cluster velocities and times for traversing intervals

The velocity of an n-cluster is given by the mean force acting on it times the mobility μ. In the scaled variables, this means that an n-cluster at position y has the velocity

$$u_n(y) = \frac{1}{n}\sum_{j=0}^{n-1}\big(f+\sin[2\pi(y+j\sigma)]\big) = f + \frac{\sin(\pi n\sigma)}{n\sin(\pi\sigma)}\sin[2\pi y + \pi(n-1)\sigma]\,. \tag{8}$$

The time needed for an n-cluster to traverse an interval $[a,b]$ with $u_n>0$ is

$$\tau_n(a,b) = \int_a^b \frac{\mathrm{d}y}{u_n(y)} = \begin{cases} \dfrac{C_n}{\pi}\arctan\Big(C_n\dfrac{\sin(\pi n\sigma)}{n\sin(\pi\sigma)} + C_n f\tan[\pi y + \pi(n-1)\sigma/2]\Big)\Big|_a^b\,, & f^2n^2\sin^2(\pi\sigma) > \sin^2(\pi n\sigma),\\[2ex] -\dfrac{C_n}{\pi}\operatorname{artanh}\Big(C_n\dfrac{\sin(\pi n\sigma)}{n\sin(\pi\sigma)} + C_n f\tan[\pi y + \pi(n-1)\sigma/2]\Big)\Big|_a^b\,, & f^2n^2\sin^2(\pi\sigma) < \sin^2(\pi n\sigma)\,,\\[2ex] \dfrac{1}{f\pi\,[1+\cot(\pi y + \pi(n-1)\sigma/2)]}\Big|_a^b\,, & f^2n^2\sin^2(\pi\sigma) = \sin^2(\pi n\sigma)\,, \end{cases} \tag{9a}$$

where

$$C_n = \sqrt{\frac{n^2\sin^2(\pi\sigma)}{\left|f^2n^2\sin^2(\pi\sigma) - \sin^2(\pi n\sigma)\right|}}\,. \tag{9b}$$

Suppl. Note 3. Soliton types

Solving Eq. (4) subject to the force conditions [Eq. (2) in the main text] and an initial condition with one double-occupied potential well, we find that after a transient time, periodic motions of two soliton types are possible: an A type given by two subintervals of the movements of an $(n+1)$- and n-cluster during one period [$(n+1)$-n-soliton], and a B type given by four subintervals of cluster movements [$(n+1)$-n-$(n+1)$-$(n+2)$-soliton], see Fig. **1**. We define n as the core size of a soliton. The decrease and increase of a cluster size in the sequences is by detachments and attachments of a single particle to the cluster [1].

Within one period of motion of an A type soliton with core size n, a single particle detaches at the back end of an $(n+1)$-cluster and attaches at the front end of an n-cluster. Back and front end refer to the direction of cluster motion in the corotating frame, i.e. to the direction of positive x for $f>0$. Within one period of motion of a B type soliton with core size n, a single particle first detaches and attaches as for the A type soliton, and in addition the detached single particle reattaches at the back end of an $(n+1)$-cluster and thereafter detaches again.

We denote by y_k the positions of a cluster (position of its leftmost particle) at the time instants of the detachment and attachment events, where k labels the corresponding event, i.e. $k=1,2$ for the A type, and $k=1,2,3,4$ for the B type (see also Fig. **1**). These positions y_k depend on the core size n of the soliton, but here and in the following we do not indicate this dependence.

At the position y_1, a single particle detaches from an $(n+1)$-cluster at its back end. At this moment, the particles of the $(n+1)$-cluster have coordinates $y_1, y_1+\sigma, \ldots, y_1+n\sigma$. The detachment can take place between any two optical traps along the ring. We set $y_1\in[0,1[$. The condition on the external forces for the detachment yields

$$f+\sin(2\pi y_1) = f + \frac{1}{n}\sum_{j=1}^{n}\sin[2\pi(y_1+j\sigma)] = f + \frac{\sin(\pi n\sigma)}{n\sin(\pi\sigma)}\sin\left(2\pi y_1 + \pi(n+1)\sigma\right)\,. \tag{10}$$

Solving for $y_1\in[0,1[$, we obtain

$$y_1 = \frac{1}{2} + \frac{1}{2\pi}\operatorname{arccot}\left[\frac{n\sin(\pi\sigma)}{\sin(n\pi\sigma)\sin[\pi(n+1)\sigma]} - \cot[\pi(n+1)\sigma]\right]\,, \tag{11}$$

where $\operatorname{arccot}(z)\in\,]0,\pi/2]$ for $z\geq 0$ and $\operatorname{arccot}(z)\in\,]-\pi/2,0[$ for $z<0$.

For both soliton types, the remaining n-cluster propagates until it attaches to the single particle resting at the position y_n^{eq}. Said differently, the single particle at the resting position y_n^{eq} attaches at the front end of the n-cluster. The position y_2 of both the n-cluster and the $(n+1)$-cluster at the time instant of the attachment is

$$y_2 = y_n^{\mathrm{eq}} - n\sigma = \frac{1}{2} + \frac{1}{2\pi}\arcsin(f) + n(1-\sigma)\,. \tag{12}$$

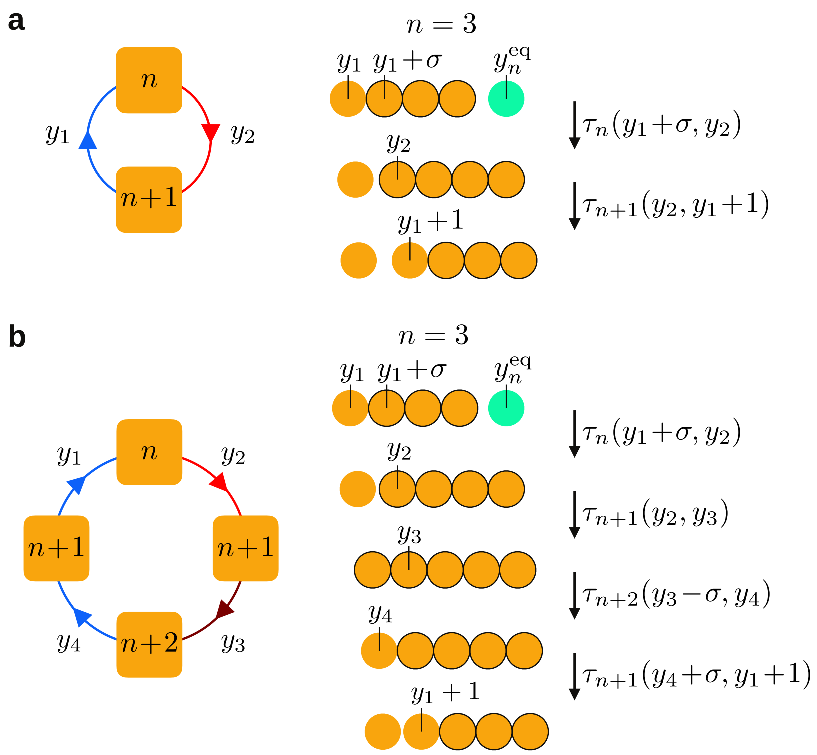

Suppl. Fig. 1. **Illustration of soliton types.** Propagation of solitons is mediated by clusters of particles in contact, where the cluster size changes by detachments and attachments of single particles. Detachment events are marked by blue arrows and occur at the back end of clusters (back and front end refer to the cluster's direction of movement). Attachment events are marked by red arrows, where bright (dark) red arrows indicate attachments at the front (back) end of a cluster. Framed circles represent those particles that keep in contact with progressing time. Two types of solitons occur: (**a**) Type A soliton with core size n consisting of subsequent movements of an $(n{+}1)$- and n-cluster during one period. The $(n{+}1)$-cluster changes to an n-cluster due to the detachment of a single particle, if the $(n{+}1)$-cluster is at position $y_1 \in [0,1[$ (the position of a cluster is always defined by the position of its leftmost particle). The n-cluster propagates until attaching to the resting particle at position $y_n^{\rm eq}$, by which a new $(n{+}1)$-cluster is generated. Both the n- and $(n{+}1)$-cluster are at position $y_n^{\rm eq} - n\sigma$ at the moment of the attachment. The newly formed $(n{+}1)$-cluster continues to move until it reaches the position $y_1 + 1$, where one cycle of the soliton motion is finished and the process starts anew. (**b**) Type B soliton with core size n consisting of subsequent movements of an $(n{+}1)$- n-, $(n{+}1)$- and $(n{+}2)$-cluster during one period. Starting with the detachment of a single particle from the $(n{+}1)$-cluster, analogous cluster moves occur as in the case of the type A soliton, until the attachment of the n-cluster to the resting particle at position $y_n^{\rm eq}$. The newly formed $(n{+}1)$-cluster, however, now continues to move until the previously detached single particle reattaches at its back end. This happens when the $(n{+}1)$-cluster is at position y_3 and, accordingly, the single particle at position $y_3 - \sigma$. The $(n{+}2)$-cluster formed by the reattachment moves until reaching position y_4, where the particle at its back end detaches again. The remaining $(n{+}1)$-cluster moves until reaching position $y_1 + 1$, by which the soliton cycle is completed. The duration of the cluster movements is given by the functions $\tau_n(a,b)$ in Eq. (9a), where a and b denote the initial and final position of the cluster [1].

Note that by the subtraction of $n\sigma$ from $y_n^{\rm eq}$ the position of the leftmost particle in the $(n{+}1)$-cluster is obtained, see Fig. **1**. For the type A soliton, the generated $(n{+}1)$-cluster moves until one period is finished, i.e. until it reaches the position $y_1 + 1$.

For the type B soliton, the $(n+1)$-cluster slows down its motion after it was formed by the attachment of the n-cluster to the resting particle. The particle having detached before from the n-cluster is reattaching to the $(n{+}1)$-cluster when this cluster is at position y_3. The determining equation for y_3 is obtained by requiring the duration of the single particle movement and that of the successive movements of the n- and $(n{+}1)$-clusters to be equal,

$$\tau_1(y_1, y_3 - \sigma) = \tau_n(y_1 + \sigma, y_2) + \tau_{n+1}(y_2, y_3)\,. \tag{13}$$

As for the arguments of the functions $\tau_n(.,.)$, note that the detached particle starts its motion at y_1 and has the position $y_3 - \sigma$ at the moment of the reattachment, see Fig. **1**. The n-cluster has finished its motion after passing the interval between positions $y_1 + \sigma$ and y_2, and the $(n{+}1)$-cluster starts its motion at y_2 and has position y_3 at

the moment of the reattachment. Inserting the functions $\tau_n(.,.)$ from Eq. (9a) gives a transcendental equation for y_3, which can be solved, e.g., by using the iterative Newton-Raphson method.

After the reattachment of the single particle, the generated $(n+2)$-cluster propagates until its leftmost particle detaches again, when the $(n+2)$-cluster is at position y_4. The condition on the external forces for the detachment give the result analogous to Eq. (11), with n replaced by $(n+1)$,

$$y_4 = \frac{1}{2} + \frac{1}{2\pi} \operatorname{arccot}\left[\frac{(n+1)\sin(\pi\sigma)}{\sin[(n+1)\pi\sigma]\sin[\pi(n+2)\sigma]} - \cot[\pi(n+2)\sigma]\right] . \tag{14}$$

Suppl. Note 4. Cycle duration, mean velocity, and mean size of solitons, duration of cluster movements

The cycle duration time τ_n^{A} for a type A soliton of core size n is the sum of the times of the motion of the n- and $(n+1)$-cluster,

$$\tau_n^{\mathrm{A}} = \tau_n(y_1+\sigma, y_2) + \tau_{n+1}(y_2, y_1+1) . \tag{15}$$

Let us note again here that the y_k are dependent on n. Analogously, the cycle duration time τ_n^{B} for a type B soliton of core size n is the sum of the times of the motion of the n-, $(n+1)$-, $(n+2)$- and $(n+1)$-cluster,

$$\tau_n^{\mathrm{B}} = \tau_n(y_1+\sigma, y_2) + \tau_{n+1}(y_2, y_3) + \tau_{n+2}(y_3-\sigma, y_4) + \tau_{n+1}(y_4+\sigma, y_1+1) . \tag{16}$$

With these results, we can calculate the fractions of times that clusters of particular sizes propagate. These time fractions give also the probabilities p_m^{α} of finding a soliton of type α ($\alpha = \mathrm{A}, \mathrm{B}$) with core size n in a state where the motion is due to a cluster of size m ($m = n, n+1$ for a type A soliton and $m = n, n+1, n+2$ for a type B soliton with core size n). We obtain:

$$p_n^{\mathrm{A}} = \frac{\tau_n(y_1+\sigma, y_2)}{\tau_n^{\mathrm{A}}} , \quad p_{n+1}^{\mathrm{A}} = \frac{\tau_{n+1}(y_2, y_1+1)}{\tau_n^{\mathrm{A}}} , \tag{17}$$

$$p_n^{\mathrm{B}} = \frac{\tau_n(y_1+\sigma, y_2)}{\tau_n^{\mathrm{B}}} , \quad p_{n+1}^{\mathrm{B}} = \frac{\tau_{n+1}(y_2, y_3) + \tau_{n+1}(y_4+\sigma, y_1+1)}{\tau_n^{\mathrm{B}}} , \quad p_{n+2}^{\mathrm{B}} = \frac{\tau_{n+2}(y_3-\sigma, y_4)}{\tau_n^{\mathrm{B}}} . \tag{18}$$

These probabilities are presented in Fig. 2(c) of the main text.

In the dimensionless units of the scaled variables, the mean velocity $\bar{u}_n^{\alpha}$ of a soliton of type α ($\alpha = \mathrm{A}, \mathrm{B}$) with core size n is

$$\bar{u}_n^{\alpha} = \frac{1}{\tau_n^{\alpha}} . \tag{19}$$

In the corotating frame, the mean angular velocity $\bar{\omega}_n^{\alpha\,\prime} = \bar{v}_n^{\alpha\,\prime}/R$ is

$$\bar{\omega}_n^{\alpha\,\prime} = \frac{\bar{v}_n^{\alpha\,\prime}}{R} = \frac{\mu\pi U_0}{\lambda R\, \tau_n^{\alpha}} , \tag{20}$$

and in the laboratory frame, $\bar{\omega}_n^{\alpha} = \bar{\omega}_n^{\alpha\,\prime} - \omega$.

The mean size $\langle n\rangle_{\alpha}$ of a soliton of type α with core size n is

$$\langle n\rangle_{\mathrm{A}} = np_n^{\mathrm{A}} + (n+1)p_{n+1}^{\mathrm{A}} = n + \frac{\tau_{n+1}(y_2, y_1+1)}{\tau_n^{\mathrm{A}}} , \tag{21a}$$

$$\langle n\rangle_{\mathrm{B}} = np_n^{\mathrm{B}} + (n+1)p_{n+1}^{\mathrm{B}} + (n+2)p_{n+2}^{\mathrm{B}} = n + \frac{\tau_{n+1}(y_2, y_3) + \tau_{n+1}(y_4+\sigma, y_1+1) + 2\tau_{n+2}(y_2-\sigma, y_4)}{\tau_n^{\mathrm{B}}} . \tag{21b}$$

Figure **2** shows (a) the mean size $\langle n\rangle$ and (b) the mean angular velocity $\bar{\omega}'$ of solitons as a function of the particle diameter σ. Note that the core size n and the type of the soliton changes with σ, which leads to the kinks of the functions plotted in Figs. **2**(a) and (b). Remarkably, the equations (5) and (6) of the main text, which were obtained from intuitive reasoning and give $\langle n\rangle \sim \bar{\omega}'/\omega \sim 1/(1-\sigma/\lambda)$, provide a good approximate description of the functional dependence on σ.

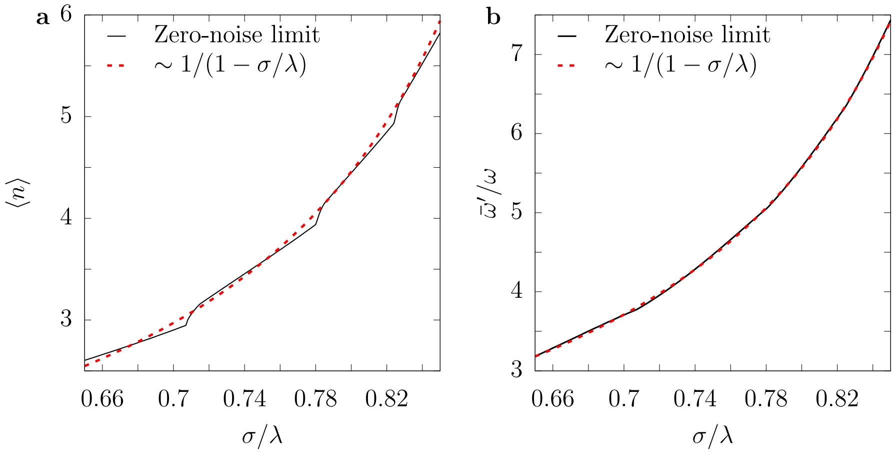

Suppl. Fig. 2. **Mean sizes and mean velocities of solitons in the zero-noise limit for $f = 0.67$.** (**a**) Mean size $\langle n \rangle$ of solitons as a function of particle diameter σ. (**b**) Normalized mean angular velocity $\bar{\omega}'/\omega$ in the corotating frame in dependence of σ. The black solid lines mark the exactly calculated results in the zero-noise limit, and the dashed red lines the approximate functional behavior according to equations (5) and (6) of the main text.

SUPPLEMENTARY REFERENCES

---

[1] Cereceda-López, E. Non-Equilibrium Dynamics of Driven and Confined Colloidal Systems. (PhD thesis, University of Barcelona, 2023), http://hdl.handle.net/10803/688857.
