# Supplementary material for: Overcrowding induces fast colloidal solitons in a slowly rotating potential landscape
Source: Nat Commun. 2023 Oct 13;14:6448. doi: 10.1038/s41467-023-41989-x (PMC10575966; doi:10.1038/s41467-023-41989-x)
Supplement: Supplementary file 3 — Description of Additional Supplementary Files [file 41467_2023_41989_MOESM3_ESM.pdf]

Description of Additional Supplementary Information

Title: Supplementary Movie 1

Description: Motion of N = 27 colloidal particles with diameter σ = 4 μm that are dragged by M = 27 optical traps. The optical traps are arranged along a ring of radius R = 20 μm that rotates at constant angular velocity ω = 0.36 rad s^{-1}. The motion of the traps corresponds to a traveling-wave potential with wavelength λ = 4.7 μm and the particles stay in the wells of this potential. No soliton is present in the system. Particle trajectories extracted from this movie are shown in the left column of Figure 1d.

Title: Supplementary Movie 2

Description: Soliton emerging in the overcrowded system (N = M + 1 = 28; other parameters are as in Supplementary Movie 1. The soliton is propagating along the ring against the direction of the rotating optical traps. It is composed of a cluster of 5 - 6 colloidal particles that periodically exchange position with their neighbors. A sequence of images taken from this movie is shown in Figure 1b, and particle trajectories extracted from it are depicted in Figure 1d, right column.

Title: Supplementary Movie 3
Description: A 3 - 2 soliton propagating along the ring of optical traps for σ/λ = 0.67. A sequence of images taken from this movie is shown in the top row of Figure 2a.

Title: Supplementary Movie 4
Description: A 4 - 3 soliton propagating along the ring of optical traps for σ/λ = 0.76. A sequence of images taken from this movie is shown in the bottom row of Figure 2a.

Title: Supplementary Movie 5
Description: Interaction of three solitons for σ/λ = 0.67. At overcrowding N - M = 3, three stable solitons propagate along the ring against the direction of the rotating optical traps. They tend to keep a constant mean distance between each other, indicating an effective repulsion between the solitons. An image from this movie is shown in the leftmost column, second top row of Figure 4a. The distribution of phase differences between pairs of solitons is shown in Figure 4c.
